# Health related quality of life in sickle cell patients: The PiSCES project

**DOI:** 10.1186/1477-7525-3-50

**Published:** 2005-08-29

**Authors:** Donna K McClish, Lynne T Penberthy, Viktor E Bovbjerg, John D Roberts, Imoigele P Aisiku, James L Levenson, Susan D Roseff, Wally R Smith

**Affiliations:** 1 Department of Biostatistics, Virginia Commonwealth University, Richmond, VA, USA; 2 Division of Quality Health Care, Department of Medicine, Virginia Commonwealth University, Richmond, VA, USA; 3 Department of Health Evaluation Sciences, University of Virginia, Charlottesville, VA, USA; 4 Division of Hematology/Oncology, Department of Medicine, Virginia Commonwealth University, Richmond, VA, USA; 5 Department of Emergency Medicine, Virginia Commonwealth University, Richmond, VA, USA; 6 Department of Psychiatry, Virginia Commonwealth University, Richmond, VA, USA; 7 Department of Pathology, Virginia Commonwealth University, Richmond, VA, USA

## Abstract

**Background:**

Sickle cell disease (SCD) is a chronic disease associated with high degrees of morbidity and increased mortality. Health-related quality of life (HRQOL) among adults with sickle cell disease has not been widely reported.

**Methods:**

We administered the Medical Outcomes Study 36-item Short-Form to 308 patients in the Pain in Sickle Cell Epidemiology Study (PiSCES) to assess HRQOL. Scales included physical function, physical and emotional role function, bodily pain, vitality, social function, mental health, and general health. We compared scores with national norms using t-tests, and with three chronic disease cohorts: asthma, cystic fibrosis and hemodialysis patients using analysis of variance and Dunnett's test for comparison with a control. We also assessed whether SCD specific variables (genotype, pain, crisis and utilization) were independently predictive of SF-36 subscales, controlling for socio-demographic variables using regression.

**Results:**

Patients with SCD scored significantly worse than national norms on all subscales except mental health. Patients with SCD had lower HRQOL than cystic fibrosis patients except for mental health. Scores were similar for physical function, role function and mental health as compared to asthma patients, but worse for bodily pain, vitality, social function and general health subscales. Compared to dialysis patients, sickle cell disease patients scored similarly on physical role and emotional role function, social functioning and mental health, worse on bodily pain, general health and vitality and better on physical functioning. Surprisingly, genotype did not influence HRQOL except for vitality. However, scores significantly decreased as pain levels increased.

**Conclusion:**

SCD patients experience health related quality of life worse than the general population, and in general, their scores were most similar to patients undergoing hemodialysis. Practitioners should regard their HRQOL as severely compromised. Interventions in SCD should consider improvements in health related quality of life as important outcomes.

## Background

Functional status and health-related quality of life (HRQOL) may be impaired in sickle cell disease (SCD) due to morbid events, such as stroke, or other organ system failures. The Cooperative Study of Sickle Cell Disease (CSSD) found that morbid events such as strokes that impaired function often preceded death in childhood [1-3] Until recent decades, SCD was associated with chronic childhood pain, organ failure and death in very early adulthood. Treatment advances have now transformed SCD into a chronic disease suffered by children and adults. Frequently, patients surviving until adulthood experience significant organ system damage that may include stroke, pulmonary failure and pulmonary hypertension, renal failure, congestive heart failure, leg ulcers, and osteonecrosis of the femoral or humeral heads [2].

Children and adolescents with SCD report poor HRQOL in qualitative studies using focus groups [4], and fare worse in their HRQOL compared to controls on health surveys [5] or on assessments of general physical, motor and independent daily functioning [6,7]. Despite the considerable evidence in children for reduced HRQOL in SCD, few studies have evaluated the impact of this disease on health related quality of life in adults [8-11].

The impact of this disease on HRQOL for adults may be even greater than for children. Quality of life is deteriorated by episodic, debilitating pain associated with substantial analgesic use, frequent hospitalization for pain episodes, and ultimately organ failure. Although SCD related pain can often be managed by analgesics and opioids, adults with SCD may be under-treated because clinicians suspect drug dependence in this population. This provider bias may lead to reluctance by patients to seek medical attention [12,13]. Further, HRQOL may be over-estimated by providers that do not regularly care for patients with SCD due to lack of understanding of the severity of the painful crises and the potential impact on function. Therefore, assessing the quality of life among a US adult SCD population and providing comparisons with HRQOL reported among comparable adults with other chronic diseases may be an important method for providing health care practitioners who care for these patients a more objective perspective on the impact and severity of this disease. In addition, quality of life in SCD is important to describe because it amplifies the ability to identify patients for whom potentially dangerous but potent interventions such as hydroxyurea or bone marrow transplantation is justified at an early stage.

## Methods

### Study description

The Pain in Sickle Cell Epidemology Study (PiSCES) is a longitudinal cohort study of over 300 adult patients with SCD designed to understand the relationship between pain and response to pain. The emphasis is on potentially mutable etiologic, and non-biologic variables. The PiSCES methods have been described in detail elsewhere [14]. Briefly, we enrolled 308 adult patients with SCD from July 2002 through August 2004. Baseline information, laboratory data and daily pain diary data were collected. Baseline data was collected at the time of enrollment using a self-administered questionnaire which included questions on demographics, health related quality of life, and other information including medical history and medication use. In addition to the survey information, blood was obtained for genotyping and urine specimens were collected to assess renal function.

As part of the PiSCES study, patients filled out daily diaries for up to 6 months [14]. The diary was modeled after the one used in the Multicenter Study of Hydroxyurea [15]. Among other things, the diary asked patients to report about the previous 24 hours: the worst sickle cell pain intensity, on a scale from 0 (none) to 9 (unbearable), whether or not they were in a sickle cell crisis, and whether they had gone for an unscheduled physician visit, ED visit or were hospitalized due to sickle cell pain.

### Patient population

Patients were solicited for enrollment from across Virginia, but focused on the Richmond and Tidewater areas of Virginia. Patients aged 16 years and older were eligible for enrollment Patients identified as potentially eligible for the study were invited and scheduled for an enrollment visit, at which time informed consent was obtained. The study, along with recruitment methods, was approved by the VCU IRB.

Interested patients were then screened using the Mini Mental Status Examination [16] to assure competency (excluded if score less than 27) and the ability to provide informed consent. Patents were compensated ten dollars for the initial visit, when blood and urine specimens are obtained, and the baseline survey was completed.

To assess the relative HRQOL in SCD patients, comparison groups from published reports representing three different cohorts of patients with chronic diseases including asthma [17], cystic fibrosis [18] and hemodialysis [19] patients were included. These comparison groups were selected to be similar in age and gender to the PiSCES cohort. The asthma sample consisted of 301 patients whose mean age was 38 and 56% of whom were female. The hemodialysis sample consisted of 1000 prevalent cases with a mean age of 58 and of whom 50% were female. Data regarding cystic fibrosis came from 223 adolescents and adult subjects participating in a validation of the quality of life instrument with a mean age of 25, and 54% female.

### Analytic variables

The Medical Outcome Study 36 item Short Form (SF-36) [20] is a generic measure of health related to functional status and well being. The survey is not disease- or age-specific and has been validated across a wide variety of age, race and disease groups, including many chronic diseases [20-22]. The SF-36 has high test-retest reliability, has been shown to predict a number of poor outcomes [23], has been compared with biological markers for their sensitivity to change in severity of chronic illness [24], and has been used as outcomes in clinical trials of chronic illness [25].

The SF-36 is multidimensional with subscales representing eight of the most important dimensions of HRQOL: physical function, physical role functions, emotional role functioning, bodily pain, vitality, general health, mental health and social function. Subscales are measured on a scale from 0 – 100 (with 0 being the worst and 100 the best score). Values are available for specific age and gender population subgroups for the US and other populations. In addition to the chronic disease samples used for comparison, the normal values for age matched males and females from the general US population are provided and compared with our study population.

Sickle cell genotype (Sβ^+ ^Thalasemia, Sß^o ^Thalasemia, SC and SS) is a known predictor of mortality and disease severity. The CSSCD evaluated the natural history of 3578 patients ranging in age from newborns to age 66. Hospital utilization due to pain varied according to genotype (SS = 0.8 episodes/pt/yr., Sß^o ^Thalasemia = 1.0 episodes/pt/yr., SC and Sß^+ ^Thalasemia = 0.4 episodes/pt/yr) [1]. Genotype was also a predictor of the age at death [2]. Further, among patients over the age of 20, the hospital utilization rate due to pain was correlated with mortality over the years [2]. For this study, genotype was obtained either directly from the blood specimen obtained from the patient at enrollment or from the patient's medical record. Since there were few patients with Sβ^+ ^Thalasemia and Sß^o ^Thalasemia, for purposes of analyses, two groups were defined. The more severe genotype grouping included SS and Sß^o ^Thalasemia, the less severe group included SC and Sβ^+ ^Thalasemia,

We used three calculated variables from the diary for this study. Mean daily pain was calculated as the sum of the pain intensity for all diary days, divided by the total number of days the diary was completed. The percentage of days for which a crisis was marked on the diary was calculated as 100*the number of days with crisis marked, divided by the total number of days the diary was completed. Percentage of days on which there was utilization was constructed similarly, with utilization consisting of either an unscheduled clinic visit, an ED visit or an overnight hospitalization. Since these latter two variable was very skewed, with many having no crisis or utilization, for analysis the diary variables were divided into 3 categories of roughly equal size (coded 1, 2, 3): Percent of days with self-reported crises: 0, 0.1–10, 10+; Percent of days with utilization: 0, 0.1–3, 3+.

### Statistical methods

Means and standard deviations are presented. Comparison values were created from MOS national norms data by using a weighted average of age-gender specific values, with the weights equal to the proportion of the PiSCES sample in that age group. Subscales for the PiSCES cohort were compared to national norms with a t-test. Analysis of variance was used as an overall test for equality of each subscale across chronic disease cohorts. When the overall F test was significant, Dunnett's test was used to compare each of the chronic diseases to the mean for the PiSCES cohort. To determine whether SCD-specific variables were independently predictive of HRQOL, we used multiple linear regression, controlling for socio-demographic variables (age, gender, education). SCD-specific variables included genotype, mean pain, and percent days of diary days reporting crisis and utilization. These analyses were limited to patients who had returned at least 30 diaries. This reduced the sample from 308 to 226. Three additional subjects were excluded from regression analyses because they lacked information on genotype. Analysis used SAS 8.2 for UNIX.

## Results

Table 1 describes the PiSCES cohort. The mean age was 33, and ranged from 16 to 64. There were more women than men in the study (60.4% vs 39.6), 48.6% attended college. Only 21.8% of subjects were currently married.

**Table 1:** Demographic description of PiSCES cohort

**Variable**	**Frequency (percent)**
Gender	
Male	122 (39.6)
Female	186 (60.4)
Education	
<High school	41 (13.4)
High School grad	116 (37.9)
Some college	110 (35.9)
College Grad	39 (12.7)
Age group	
16–24	79 (25.6)
25–34	92 (29.9)
35–44	82 (26.9)
45–54	42 (13.6)
55–64	12 (3.9)
Marital Status	
Married	67 (21.8)
Never married	198 (64.5)
Divorced/separated/widowed	42 (13.7)
Genotype	
SS	206 (66.9)
Sß^o ^Thalasemia	8 (2.6)
SC	75 (4.3)
Sβ^+ ^Thalasemia	10 (3.2)
Unknown	9 (2.9)

Table 2 has means and standard deviations for the 8 SF-36 subscales for the PiSCES cohort, separately for men and women, along with the age-adjusted national norms by gender. When the gender stratified PiSCES cohort was compared with national norms, values were significantly lower for all subscales (P < 0.0001) with one exception – the mental health scale was not significantly different from the national norm (men: p = 0.670, women: p = 0.102).

**Table 2:** SF-36 – PiSCES cohort vs National Norms (Mean ± standard deviation)

	**Male**		**Female**	
	**PiSCES**	**Norm**	**PiSCES**	**Norm**

Physical Function	66.4 ± 24.1	92.3 ± 15.5	59.9 ± 25.1	87.4 ± 19.7
Role-Physical	40.1 ± 38.7	90.5 ± 24.0	38.6 ± 39.9	83.8 ± 31.5
Bodily Pain	50.8 ± 28.6	79.6 ± 21.1	45.2 ± 26.0	77.3 ± 22.1
General Health	42.7 ± 22.3	77.1 ± 17.3	37.0 ± 21.7	73.9 ± 19.0
Vitality	50.4 ± 22.5	64.9 ± 19.2	37.6 ± 21.0	59.2 ± 20.6
Social Function	62.3 ± 27.6	86.6 ± 19.9	62.4 ± 24.8	82.8 ± 22.1
Role-emotional	62.7 ± 43.1	85.0 ± 29.4	54.7 ± 42.8	80.7 ± 33.1
Mental Health	75.3 ± 20.7	76.4 ± 16.8	69.2 ± 20.0	72.8 ± 18.3

P < 0.0001 comparing PiSCES to SF-36 for all subscales, except MH, female: p = 0.102 and male: p = 0.670;

Table 3 has means and standard deviations of the 8 SF-36 subscales for the PiSCES cohort along with cohorts of patients with three other chronic diseases – asthma, cystic fibrosis and hemodialysis. There were significant differences (P < 0.0001) amongst cohorts for all subscales except mental health (p = 0.0582), which was marginal. Patients with SCD (PiSCES cohort) reported significantly worse HRQOL on all subscales (p < 0.05) except mental health as compared to adolescents and adults with cystic fibrosis. They had similar reported quality of life as asthma patients regarding physical function and role function (both physical and emotional), and mental health, but scored lower for the bodily pain, vitality, social function and general health subscales. When compared to patients on hemodialysis, SCD patients reported similar low scores for physical and emotional role function and social function. They also did not differ on the mental health subscale (p > 0.15). SCD patients had lower scores for the pain, vitality and general health subscales (P < 0.01), but reported a higher score for the physical function subscale compared with the hemodialysis cohort.

**Table 3:** SF-36: Comparison of PiSCES sample with other chronic disease cohorts (mean ± standard deviation)

	**PiSCES**	**Hemo-Dialysis**	**Cystic Fibrosis**	**Asthma**	**ANOVA F value^†^**
	N = 308	N = 1000	N = 223	N = 241	

Physical Function	62.4 ± 24.9	44.3 ± 27.8*	76.3 ± 24.0*	63.2 ± 21.4	121.3
Role-Physical	39.2 ± 39.4	39.7 ± 40.4	72.9 ± 38.4*	38.7 ± 39.9	74.7
Bodily Pain	47.4 ± 27.2	60.4 ± 29.1*	82.2 ± 21.3*	67.2 ± 23.2*	49.5
General Health	39.2 ± 22.1	50.0 ± 22.4*	43.4 ± 23.7*	57.9 ± 19.0*	37.9
Vitality	42.7 ± 22.5	46.5 ± 22.3*	58.4 ± 23.1*	48.2 ± 20.8*	23.3
Social Function	63.5 ± 25.2	66.0 ± 29.9	80.4 ± 23.8*	72.1 ± 22.2*	21.3
Role-emotional	57.8 ± 43.1	58.2 ± 42.7	77.0 ± 36.9*	63.3 ± 41.5	13.1
Mental Health	71.6 ± 20.4	69.7 ± 21.6	73.7 ± 18.1	70.7 ± 18.4	2.56

^†^Numerator degrees of freedom are 3, denominator degrees of freedom are N-4;

*p < 0.0001 compared to PiSCES cohort

Multiple regressions were performed to look at the relationship of SCD specific variables (genotype, mean pain, percent of diary days subjects reported crisis and percent of diary days subjects reported utilization) and SF-36 subscales. Socio-demographic variables (age, gender, number of years of education) were included in the models as covariates. Results are in Table 4. For each subscale, mean SCD pain was highly predictive (p < 0.0001 for all subscales except p = 0.0396 for mental health). The more SCD pain a subject experienced, the worse the reported quality of life. One unit increase in pain (on a 0–9 scale) was associated with an approximate decrease of 1.4 (mental health) to 6 (both role functions) units on an SF-36 subscale. Percentage of days with crisis was an independent predictor of bodily pain (p = 0.0109), with an approximate 6 point decrease in bodily pain score for each increase in crisis category. Genotype was also an independent predictor of vitality (p = 0.0161), with SS/Sß^o ^Thalasemia being associated with better vitality. No other variables were independent predictors of SF-36 subscales.

**Table 4:** Results of regression of SCD-specific variables on SF-36 subscales, controlling for socio-demographic variables^1 ^(regression coefficients ± standard error)

	**Genotype^2^**	**Mean pain**	**Proportion Days with Crisis^3^**	**Proportion Days with Utilization^4^**
Physical Function	-5.01 ± 3.30	-4.55 ± 0.72**	-0.42 ± 2.12	3.51 ± 1.97
Role-Physical	-1.62 ± 5.73	-6.12 ± 1.27**	-0.91 ± 3.70	2.75 ± 3.39
Bodily Pain	0.14 ± 3.57	-4.41 ± 0.79**	-5.93 ± 2.31*	-0.59 ± 2.13
General Health	3.52 ± 3.19	-3.34 ± 0.71**	-1.31 ± 2.09	-1.44 ± 1.92
Vitality	6.43 ± 3.27*	-3.54 ± 0.72**	0.55 ± 2.10	3.06 ± 1.94
Social Function	3.16 ± 3.77	-4.27 ± 0.82**	-3.28 ± 2.44	1.93 ± 2.25
Role-emotional	7.06 ± 6.27	-5.81 ± 1.39**	2.30 ± 4.09	2.12 ± 3.71
Mental Health	4.23 ± 3.14	-1.44 ± 0.69*	-1.53 ± 2.02	0.35 ± 1.87

^1^controlling for age, gender, years of education

^2^SS and Sß^o ^Thalasemia vs SC and Sß^+ ^Thalasemia

^3^Percent of days with crises: 0, 0.1–10,10+ (coded 1,2,3)

^4^Percent of days with utilization: 0, 0.1–3, 3+ (coded 1,2,3)

*p ≤ 0.05; **p < 0.0001

## Discussion

In general, SCD patients experience a poor health related quality of life. Except for mental health, the SF-36 subscale values were considerably lower than norms of the general US population. They reported a HRQOL that was equal to or poorer than patients with other significant chronic conditions in many domains. Similar to patients with SCD, until somewhat recently, patients with cystic fibrosis rarely lived until adulthood, marking this as a disease with significant sequelae and high mortality. It is interesting, then, to see that quality of life of adult survivors of this chronic disease, even though impaired, was comparable to national norms, and was generally far superior than that reported by adults with SCD. Of the three chronic conditions selected for comparison, the PiSCES cohort had HRQOL patterns most similar to that of patients undergoing chronic hemodialysis.

That SCD patients did not report poorer mental health and well being than the general US population is consistent with findings for many medical conditions. In 1978 Brickman, et al [26] presented a famous result showing that lottery winners are not much happier than paraplegics. Since that time many studies have confirmed similar results, that while people who are sick may report their health as being worse than the general population, they appear to have similar well-being. Not only was it true of the asthma, dialysis and cystic fibrosis cohorts presented here, but similar results have been shown for people with other chronic diseases both in the US and other countries [21,22,27].

It has been suggested that the fact that many people with chronic diseases report good psychological well being could be a result of increased social support, lack of other stressors, or a "response shift" associated with the managing their chronic disease [27]. The "response shift" could be a result of a scale recalibration, a change in the patient's values, or a reconceptualization of their mental health and well-being [28,29] in order to accommodate their illness. Riis et al [30] dispute the idea of scale recalibration, proposing instead that people have adapted to their illness or situation.

Heady and Wearing [31] propose that there is a baseline level of mood or well-being that people have to which they return after events cause them to move from that baseline. This is supported by a twin study indicating that most variation in well-being is due to variations in genetics, not life circumstances [32]. That would suggest that, while perhaps people with SCD may temporarily report poorer well-being associated with high levels of pain or other disease sequalae, most often they would report a baseline well-being similar to that of others.

Despite the similar level of well-being in SCD patients compared with both norms and patients with other chronic diseases, patients with SCD experience significant decrements in other important aspects of HRQOL. This is supported in a study of adult SCD patients in the UK, where Anie et al, found their population also had much lower HRQOL scores than general UK population norms. Further, the patients in this study had reported HRQOL similar to patients with arthritis due to hereditary haemochromatisis, another chronic disease [9]. Patients in the PiSCES cohort reported somewhat lower general health and higher mental health scores than Anie et al found. This may result in part from differences in the two cohorts, including their relative access to health care in the two settings.

Surprisingly, HRQOL was not associated with genotype except for the vitality subscale. However there was a strong association with reduced HRQOL and pain levels, and, for a few subscales, there was a trend with increasing levels of utilization. The relationship between genotype severity and HRQOL may have been mediated by these variables, particularly pain. Anie et al [9] also found a relationship between pain and some subscales of the SF-36 (physical and social functions, mental and general health) but found no significant association with utilization measures. Whether the more severe manifestations of the disease cause the poorer quality of life, or patients who report poorer quality of life suffer more and use more health care cannot be determined from this study.

It is unclear why the direction of the statistically significant association between vitality for SS/Sß^o ^Thalasemia vs SC/Sß^+ ^Thalasemia is opposite of what would be expected, with the more severe genotype being associated with better vitality. Even the nonsignificant relationships between genotype and the other HRQOL subscales in these regressions were in the same direction, except for physical functioning. Similarly, increased utilization tended to be associated, albeit nonstatistically with better HRQOL scores for most subscales These counter-intuitive findings should be explored by other SCD researchers.

There are several limitations of this study. First, this study enrolled patients from only one state, so may not be representative of the entire US SCD population. When comparing to populations with chronic disease, the gender and age distributions were not ideally matched. In particular, the PiSCES cohort, at a mean age of 33, is significantly younger than the dialysis comparison group However, since SF-36 subscale scores tend to decrease with age, the fact that these younger patients with SCD had worse HRQOL scores on some subscales than an older hemodialysis cohort is even more alarming

## Conclusion

Practitioners caring for adult SCD patients should regard their quality of life as severely compromised, with scores that are most similar to hemodialysis patients in our comparison of other chronic diseases. Although reducing mortality is of paramount importance among SCD patients, future interventions should consider improving health related quality of life as a clinical endpoint.

## Authors' contributions

DKM participated in the design and coordination of the study, performed the statistical analysis, interpreted results and drafted the manuscript. IPA, SDR, JDR participated in the coordination of the study, and helped to edit the manuscript. WRS, VEB, JLL, LTP conceived of the study, participated in its design and coordination, and helped to edit the manuscript. All authors read and approved the final manuscript.
